# Investigating the causal role of motor brain areas in rhythm reproduction: a transcranial direct current stimulation study

**DOI:** 10.1038/s41598-025-02316-0

**Published:** 2025-05-23

**Authors:** Marina Emerick, Joshua D. Hoddinott, Jessica A. Grahn

**Affiliations:** 1 Centre for Brain and Mind, University of Western Ontario, London, Ontario, Canada; 2 Neuroscience, Schulich School of Medicine and Dentistry, University of Western Ontario, London, Ontario, Canada; 3 Psychology, Social Science, University of Western Ontario, London, Ontario, Canada

**Keywords:** Music cognition, Beat perception, Transcranial direct current stimulation, Supplementary motor area, Cerebellum, Premotor cortex, Psychology, Neuroscience, Cognitive neuroscience

## Abstract

Humans have an intrinsic tendency to move to the beat in music, yet the neural mechanisms behind the music-movement connection remain poorly understood. Most studies to date have been correlational. In this study, we used transcranial direct current stimulation (tDCS) to explore the causal role of four brain regions commonly involved in movement timing and beat perception: the supplementary motor area (SMA), the left and right premotor cortices (PMC), and the right cerebellum. Participants received anodal, cathodal, or sham stimulation in one of the four regions on three separate days while reproducing strong-beat, weak-beat, and non-beat rhythms via finger tapping. Because the SMA is thought to play a central role in beat perception, while the premotor cortex and cerebellum are involved in general timing processes, we predicted that SMA stimulation would influence the reproduction of beat-based rhythms, while premotor and cerebellar stimulation would affect the reproduction of non-beat rhythms. As expected, reproduction accuracy depended on beat strength; strong-beat rhythms were more accurately reproduced than weak and non-beat rhythms. Unexpectedly, tDCS had no effect on reproduction accuracy in any brain region. Thus, we found no evidence that modulating brain excitability in SMA, PMC, or cerebellum altered the accuracy of rhythm reproduction.

## Introduction

Humans spontaneously synchronize movements to music. Although some movements, like dancing and playing a musical instrument, are complex, others, like foot tapping and nodding heads, occur spontaneously and without training^1^. Spontaneous synchronization to music aligns with the steady pulse, or “beat” underlying the regular rhythm. While rhythm can be defined as “the serial pattern of variable note durations in a melody”^2^, the feeling of a recurring pattern of salient pulses is defined as the beat^3^.

The behavioural link between rhythm and the motor system has been supported by neuroimaging studies of auditory rhythm^4–11^. In addition to auditory sensory regions, rhythm perception activates motor areas, including the supplementary motor area (SMA), premotor cortices (PMC), the basal ganglia, and the cerebellum, even in tasks requiring no movement^4–7,12^. When rhythms have a clear (or strong) beat, the basal ganglia and SMA show greater activation than when rhythms have no beat^5,7,8,13^. While the SMA and basal ganglia seem to be beat sensitive, other regions in the rhythm-perception network, such as the PMC, prefrontal cortex, inferior parietal lobule, and cerebellum, do not seem to be as sensitive to the beat. These regions also exhibit greater activity for complex rhythms, in which the beat is difficult to detect^8^. It has been proposed that the SMA and dorsal striatum are responsible for structuring beat-based temporal anticipation and auditory expectations, creating a loop that facilitates beat perception through the entrainment of neural activity and auditory-motor interactions^14,15^.

Neuropsychological work with Parkinson’s disease (PD) patients has highlighted the importance of the basal ganglia in beat perception, as PD patients can serve as a model for basal ganglia dysfunction. Compared to healthy controls, PD patients perform worse than controls in discriminating strong-beat rhythms, but not weak-beat rhythms, indicating the role of the basal ganglia in beat perception^16^. In contrast, patients with cerebellar degeneration show impairments in absolute timing, which involves encoding the durations of time intervals^17,18^, but perform similarly to healthy controls in beat-based timing tasks^19^. Other studies support that the cerebellum appears to respond and encode temporal intervals that do not have a reference interval, such as a regular beat^18,20,21^. Overall, these studies support a dissociation between the basal ganglia in beat-based, or relative, timing and the cerebellum in absolute timing.

Despite extensive research in rhythm perception using correlational neuroimaging methods^5,8,18,22^ and neuropsychological studies^16,19^, few studies have used causal methods in healthy humans^23^. Transcranial direct current stimulation (tDCS) is one way to causally examine the role of different brain areas in different timing processes. TDCS modulates the synaptic efficacy of neurons by altering resting membrane potential by passing a weak electric current between two brain areas^24^, and it can modulate brain responses in two directions: anodal stimulation increases cortical excitability, while cathodal stimulation inhibits cortical excitability^25,26^. While tDCS does not directly trigger action potentials, it brings neural populations closer to or further from activation threshold, increasing or decreasing the likelihood of neural activity, respectively. Importantly, tDCS is functionally specific – behavioural changes can be seen if a modulated network is task-relevant^27^.

A previous study using strong- and weak-beat rhythms demonstrated the SMA’s crucial role in rhythm discrimination using tDCS^23^. During anodal stimulation of the SMA, participants discriminated rhythm changes more accurately than during sham stimulation, and during cathodal stimulation, participants discriminated more poorly than during sham. This polarity-dependent response is strong evidence for SMA’s role in rhythm discrimination; however, the effect was observed for both strong- and weak-beat rhythms, suggesting SMA’s contribution did not depend on beat strength. Premotor cortex stimulation elicited no consistent effect on discrimination performance. For the cerebellum, both anodal and cathodal stimulation worsened discrimination performance. These results indicate that both the SMA and cerebellum play roles in rhythm discrimination, but they do not support a selective role of the SMA in beat-based timing, as stimulation affected discrimination of both strong- and weak-beat rhythms^23,28^. Further, the cerebellar stimulation had a selective effect on strong-beat rhythm discrimination, counter to its proposed role in absolute, not relative (beat-based) timing^29^.

Here, we extend this work by employing the same stimulation methodology in a rhythm reproduction paradigm using the same stimuli as in the rhythm discrimination study. We used a rhythm reproduction task for multiple reasons. First, it can often provide a more sensitive and continuous measure of rhythm and beat perception accuracy, as well as minimize decisional effects (e.g., response bias) and serial position effects of the to-be-discriminated change in the rhythm present in perception tasks^5,8,23^. Second, the PMC group did not show tDCS effects in the prior rhythm discrimination study^23^, but it is possible that the PMC plays a role when programming rhythmic motor output. Finally, we wanted to test whether SMA stimulation would also affect performance when a motor output action was required^14,15^.

We also included non-beat rhythms along with strong- and weak-beat rhythms used previously. This enabled us to do two things. First, to determine whether the lack of difference between strong- and weak-beat rhythms for the SMA stimulation group would replicate with the non-beat rhythms^23^, which are more irregular than weak-beat rhythms. In weak-beat rhythms, a beat theoretically can be aligned to the rhythm (although, in practice, the performance of weak- and non-beat rhythms is similar unless they are presented in a looping fashion). In non-beat rhythms, no beat can be consistently aligned to the rhythm, as the irregular intervals disrupt any semblance of a beat. Second, we could examine whether cerebellar stimulation, which previously did not affect weak-beat rhythm discrimination, would be observed if more irregular (non-beat) rhythms were used.

Here, we investigated the causal role of four brain areas (SMA, right cerebellum, left PMC, and right PMC) in rhythm and beat perception through a rhythm reproduction paradigm, assessing how reproduction accuracy differed for sequences that could be timed using a beat-based versus non-beat-based timing system when neural excitability was causally altered using tDCS. A mixed design was employed because of significant individual differences in rhythm reproduction ability^9^ and tDCS responsivity^30^. Participants were randomly assigned to one of four brain area groups and completed the rhythm reproduction task on three different days while receiving sham, anodal, or cathodal tDCS stimulation.

Based on neuroimaging evidence showing the SMA is involved in the temporal processing of beat-based sequences and that the premotor cortex and cerebellum appear to respond in both beat and non-beat contexts^5^ or respond more to non-beat-based contexts^19,31^, we hypothesized that modulating excitability in the SMA should influence the ability to reproduce strong-beat rhythms more than weak- and non-beat rhythms. In contrast, if the cerebellum and PMC are involved in absolute timing, the effects of tDCS in these areas will not depend on beat strength, and their stimulation should influence accuracy for all rhythm types. It’s also possible that strong-beat timing is dissociable from weak- and non-beat timing^29,32^, in which case cerebellar and PMC stimulation may produce greater effects for weak and non-beat rhythms than strong-beat rhythms.

Findings from this study will shed light on rhythm and beat perception by providing converging evidence for a causal role of specific brain regions in beat-based and non-beat-based timing systems during rhythm reproduction. It will determine whether the SMA mediates beat-based timing in the context of a reproduction task and whether the cerebellum and PMC mediate non-beat-based timing. We are building on neuropsychological studies by stimulating areas that are not commonly dysfunctional in clinical populations, such as the SMA and PMC, using healthy controls.

## Methods

### Participants

Participants were recruited through the Western University undergraduate participant pool (SONA) or word of mouth. The Health Sciences Research Ethics Board at Western University approved the study, and the experiments were performed following relevant guidelines and regulations^33,34^.

To minimize potential risks, participants were excluded if they had a history of psychiatric or neurological problems such as epileptic seizures, Tourette’s syndrome, ADHD, depression; any metallic implants, such as pacemakers, cerebral aneurysm clips or other electronic implants; any active skin problems, such as eczema; any unstable medical condition and the susceptibility to migraine or other frequent headaches; any history of episodes of faintness; current use of a hearing aid; for female participants specifically, being or trying to become pregnant. All participants gave informed consent before beginning the experiment.

In total, 91 participants took part. Ten participants were excluded for either not completing all three sessions, feeling uncomfortable in the active session, or technical issues. Therefore, the final sample consisted of 81 participants (mean age: 19.7 ± 3.9 yrs, 54 women); 22 received SMA stimulation, 19 received right cerebellar stimulation, 19 received left PMC stimulation, and 21 received right PMC stimulation. Of the 81 participants, 74 were right-handed and 7 were left-handed. All participants were instructed to tap with the index finger of their dominant hand.

### Material

#### Questionnaires

Musical training ability was assessed with the musical training subscale from the Goldsmiths Musical Sophistication Index^35^. The subscale comprises seven items, with scores ranging from 7 to 49. A demographic questionnaire assessed educational level, language, and general health.

A questionnaire was developed following Schaal et al.^36^ to assess participants’ blindness to the type of stimulation. At the end of each session, participants indicated whether they thought they had received active or sham stimulation. If they indicated active, they indicated whether they thought it was anodal or cathodal stimulation. They also indicated how sure they were on an adapted Likert scale, with 1 = ‘completely unsure’ and 10 = ‘completely sure’. Finally, they were also asked if they noticed any sensation (e.g., tingling, itching sensation) during or after the stimulation.

#### Stimuli

Rhythm stimuli were presented using E-prime 2.0 Software (Psychology Software Tools, Pittsburgh, PA) on a Dell laptop over Bose headphones. Rhythms were generated using MATLAB (version 9.0.0, R2016a; The MathWorks Inc., Natick, MA, USA, 2016).

**Fig. 1:**
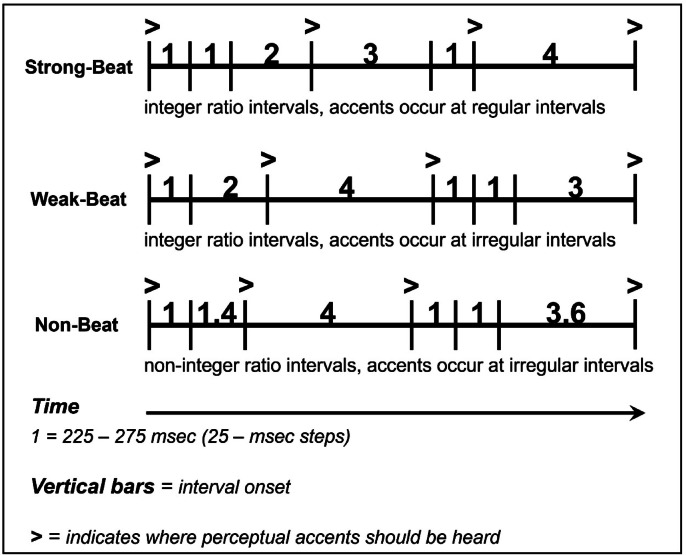
Schematic of sample stimuli. Numbers indicate the relationship between intervals. The base interval, or the shortest interval (e.g. ‘1’) ranged from 225 ms to 275 ms, in steps of 25 ms. The other intervals are multiples of the base interval; for example, ‘2’ is twice the duration of ‘1’. (Adapted from Grahn and Brett, 2007).

Stimuli comprised rhythms adapted from Grahn and Brett’s previous study^5^. Each rhythm comprised 5 to 7 intervals, and interval durations were multiples of the ‘base interval’, or shortest interval (i.e., ‘1’ interval). Base intervals could be either 225, 250 or 275 ms. For example, for a strong-beat rhythm with a base interval of 250 ms, the sequence 112,314 contains the intervals ‘250 250 500 750 250 1000’ in length (milliseconds). The base interval varied from trial to trial (but was kept consistent within a trial) in order to eliminate any carryover effects of a perceived beat rate from one trial to another. Rhythms were separated into three categories according to their beat strength: strong-beat, weak-beat, and non-beat rhythms. While strong- and weak-beat rhythms consist of integer-ratio intervals (e.g., 1:2:3:4), non-beat rhythms consist of non-integer ratio intervals, in which the ‘2’ and ‘3’ intervals are replaced by ‘1.4’ and ‘3.6’ respectively (i.e., 1:1.4:3.6:4). Strong-beat rhythms induce regularly occurring ‘perceptual accents’ at the beginning of each group of four units (e.g., ‘1111’, ‘22’, or ‘4’), emphasizing the beat at predictable intervals^21,37^. Importantly, there are no physical (e.g., intensity) accents in the stimulus—the perceptual accents arise from the interval structure alone. Weak-beat rhythms have more irregular accents and fewer tones occurring on the beat, making the beat hard to detect. Non-beat rhythms have irregular intervals in addition to irregularly spaced accents, making a consistent beat impossible (see Fig. 1).

When participants reproduce rhythms, they tap the onset of each interval. To ensure that the length of the final interval in the rhythm could be measured, each rhythm ended with an additional tone equal to the ‘1’ interval that marked the end of the final interval. For example, a six-interval rhythm would have seven tone onsets, and thus seven tap times to calculate the six interonset intervals that were the reproduced duration of those six intervals. The set of intervals used to create each rhythm was termed an ‘interval set’, and the same interval set (e.g., the interval set 11334) appeared across the three rhythm types the same number of times. There were 60 rhythms: 20 per rhythm type (see Table 1 for the complete list of rhythms).

**Table 1:** Rhythmic sequences for each condition.

	Interval Set	Strong-beat	Weak-beat	Non-Beat
5 Intervals	1 1 3 3 4	3 1 4 1 3	1 1 3 4 3	1 1 3.6 4 3.6
	1 1 3 3 4	4 1 3 3 1	3 3 1 4 1	3.6 3.6 1 4 1
	1 1 3 3 4	4 3 1 1 3	4 1 1 3 3	4 1 1 3.6 3.6
	1 2 2 3 4	2 2 4 1 3	1 3 2 4 2	1 3.6 1.4 4 1.4
	1 2 2 3 4	3 1 4 2 2	2 3 2 4 1	1.4 1 3.6 1.4 4
	1 2 2 3 4	4 3 1 2 2	4 1 2 3 2	4 1 1.4 3.6 1.4
6 Intervals	1 1 1 2 3 4	1 1 2 3 1 4	1 2 4 1 1 3	1 1.4 4 1 1 3.6
	1 1 1 2 3 4	2 1 1 4 1 3	3 2 1 4 1 1	3.6 1.4 1 4 1 1
	1 1 2 2 3 3	2 2 1 3 3 1	1 2 1 2 3 3	1 1.4 1 1.4 3.6 3.6
	1 1 2 2 3 3	3 1 1 3 2 2	2 3 1 1 2 3	1.4 3.6 1 1 1.4 3.6
	1 1 2 2 2 4	1 1 2 4 2 2	1 2 2 1 4 2	1 1.4 1.4 1 4 1.4
	1 1 2 2 2 4	2 1 1 2 2 4	2 1 4 2 2 1	1.4 1 4 1.4 1.4 1
	1 1 2 2 2 4	4 2 2 1 1 2	4 1 2 2 1 2	4 1 1.4 1.4 1 1.4
7 Intervals	1 1 1 1 1 3 4	1 1 1 1 4 3 1	1 3 1 4 1 1 1	1 3.6 1 4 1 1 1
	1 1 1 1 2 3 3	2 1 1 3 1 1 3	2 3 3 1 1 1 1	1.4 3.6 3.6 1 1 1 1
	1 1 1 1 2 3 3	3 1 2 1 1 1 3	3 1 1 3 1 2 1	3.6 1 1 3.6 1 1.4 1
	1 1 1 1 2 2 4	1 1 2 2 1 1 4	1 1 1 2 4 1 2	1 1 1 1.4 4 1 1.4
	1 1 1 1 2 2 4	2 2 1 1 1 1 4	2 1 4 1 2 1 1	1.4 1 4 1 1.4 1 1
	1 1 1 2 2 2 3	1 1 2 3 1 2 2	1 1 3 2 2 1 2	1 1 3.6 1.4 1.4 1 1.4
	1 1 1 2 2 2 3	3 1 2 2 1 1 2	3 2 2 1 1 1 2	3.6 1.4 1.4 1 1 1 1.4

Rhythmic Sequences for Each Condition. 1 = 225–275 msec (in steps of 25 msec), chosen at random for each trial. All other intervals in that sequence are multiplied by the length chosen for the 1 interval. All sequences ended with an additional ‘1’ interval, to provide the final onset that demarcates the length of the last interval in the rhythm as listed in the table.

#### Tasks

Rhythm Reproduction Task: During this task, 60 rhythms were presented in random order for each participant. Participants listened to the same rhythm three times and then reproduced the rhythm by tapping their index finger on a computer keyboard. After their response, a new rhythm was presented, until all 60 rhythms were presented. The 20 strong-beat, weak-beat, and non-beat rhythms were randomly intermixed. Before each session, participants completed four training trials with rhythms not used in the main experiment.

Self-Paced Tapping Task: To assess tDCS effects on motor output variability (independent of rhythm perception), participants completed a self-paced tapping task at the beginning of each session. Participants were asked to tap ten times in a row at a comfortable rate, while the stimulation was applied. They were told that there was no ‘right or wrong’ rate to tap.

#### Transcranial direct current stimulation (tDCS)

The Chattanooga Ionto Dual Channel Electrophoresis System was used to apply a 2-mA current over the participant’s scalp with the tDCS. Two 4 × 6 cm rubber electrodes placed in saline-soaked sponges (current density of 0.083 mA/cm^2^ in each electrode; 0.9% NaCl) were secured to the scalp with rubber head straps. For the active tDCS conditions, the current gradually ramped up to 2 mA over 30 s upon commencing the task and remained at 2 mA for the task duration of 20 min, then ramped down. For the sham tDCS conditions, the stimulation was similarly ramped up over 30 s to 2 mA but then immediately ramped back down to 0 over the next 30 s. The sham condition is sufficient to achieve blinding in stimulation-naive participants, as it evokes the sensation of being stimulated but does not lead to a neurophysiological change^38^.

**Fig. 2:**
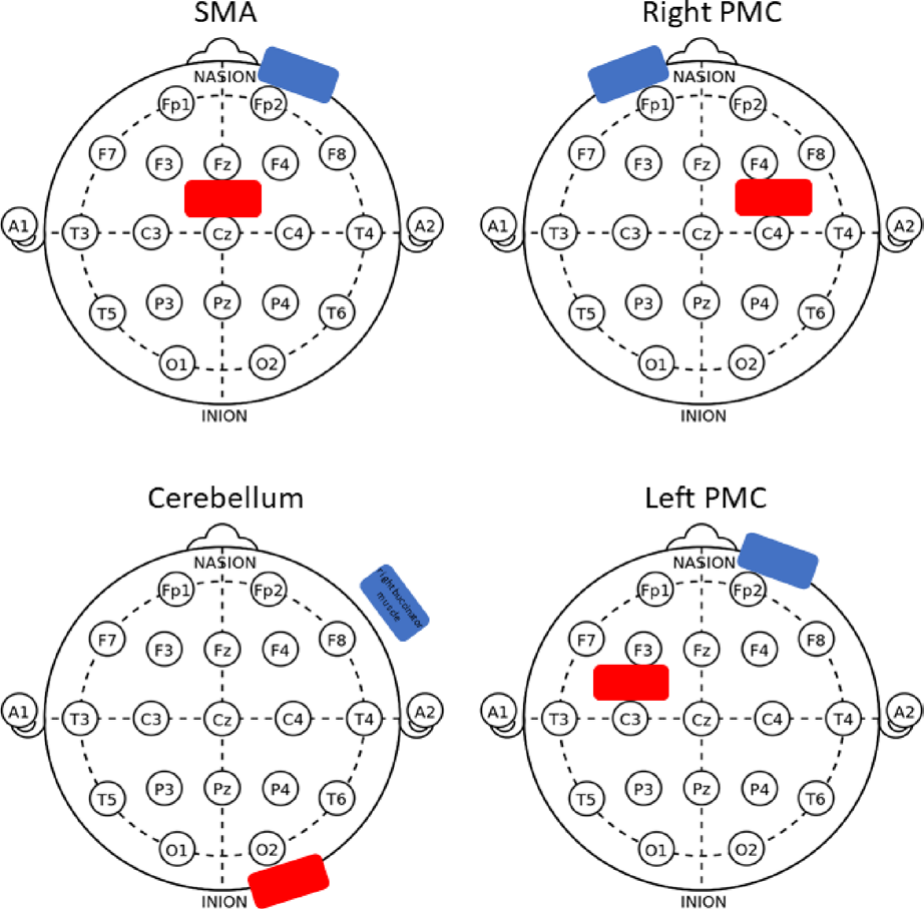
tDCS Stimulation Sites. Stimulation electrode positions, anodal electrodes in red and cathodal electrodes in blue. An anodal stimulation example is shown, as the anodal electrodes are positioned in the regions of interest, and the cathodal electrodes are positioned in the reference regions. **SMA**: anode electrode 2 cm anterior to Cz, reference above right eye forehead. **PMC**: anode electrode 2 cm anterior to C3 for right PMC, 2 cm anterior to C4 for left PMC, references in contralateral orbit. **Cerebellum**: anode 3 cm right of the inion, reference in right buccinator muscle.

The stimulation sites were located using the international electroencephalographic 10–20 system^39^ and following Leow et al. procedures^23^. As shown in Fig. 2, for the SMA site, one electrode was positioned 2 cm anterior to Cz (located at the center of the top of the head), and the other electrode was placed on the forehead above the right eye^40^; for the cerebellum, one electrode was positioned 3 cm right of the inion (at the back of the head), while the other one was positioned on the right buccinator muscle (facial muscle underlying the cheek)^41^, for the left PMC, one electrode was positioned 2 cm rostral to C3, while the other electrode was positioned on the contralateral orbit, and for the right PMC, one electrode was positioned 2 cm rostral to C4, while the other electrode was positioned on the contralateral orbit^42–44^. Anodal and cathodal stimulation were differentiated by the direction of current flow: in different sessions, the electrode over the region of interest was once the anode and once the cathode.

### Procedure

Participants completed three one-hour sessions at the University of Western Ontario, with two to seven days between sessions. The three sessions were similar in task procedure. The type of stimulation received (sham, anodal, or cathodal) was counterbalanced across sessions, and in the first session, participants completed medical screening and demographic questionnaires as well as the musical training subscale of the Goldsmiths Musical Sophistication Index.

The self-paced tapping task was completed at the beginning of each session as a control task to assess whether the tDCS stimulation interfered with tap timing. Afterward, participants completed the rhythm reproduction task. The total reproduction task lasted 20 min.

At the end of each session, participants completed the blinding questionnaire and indicated whether they believed they received active or sham stimulation. They were also told to report any different sensation they felt and that they would receive an explanation about it by the end of the study. At the end of the third day, participants were debriefed.

### Data analysis

Data was first treated using R (R Core Team, 2020), and statistical analysis was performed using JASP (JASP Team, 2022).

#### Rhythm reproduction task

##### Proportion of correct trials

Trials with the incorrect number of taps (too few or too many) were deemed incorrect and not further analyzed. For the remaining trials, the intertap time of each interval in the reproduced rhythm was compared to the corresponding interval in the presented rhythm. If any interval duration was reproduced shorter or longer than 20% of the presented interval, the trial was deemed incorrect. For each participant, the proportion of correct trials was calculated for each condition (strong-, weak-, and non-beat), and for each stimulation condition (sham, anodal, and cathodal).

##### Proportional average error

The error of reproduced intervals in correctly reproduced sequences was also analyzed. The error for each interval was calculated as the absolute difference between the reproduced interval (e.g. 250 ms) and the original interval (e.g. 300 ms), divided by the original interval (|250–300| / 300; error = 0.17). Error across all intervals in a trial was averaged, then error across all trials in each condition was averaged. The larger the average error, the worse the temporal accuracy.

Frequentist analysis: Mixed-measures ANOVAs were conducted on the proportion of correct trials and proportional average error to investigate the effects of beat strength (strong-beat vs. weak-beat vs. non-beat) and stimulation type (sham vs. anodal vs. cathodal). Brain area (SMA, right cerebellum, right PMC or left PMC) and musical experience (high vs. low) were included as the between-subject factors. Significant differences in the ANOVA were followed up with pairwise tests.

Bayesian analysis: Bayesian statistics were used to determine evidence for the null and experimental hypotheses. In contrast to the frequentist approach, the Bayesian approach quantifies support for hypotheses instead of rejecting the null hypothesis. Here, we used a Bayesian repeated mixed-measures ANOVA to select the model that best fits the data. Analysis was conducted on the proportion of correct trials to investigate the effects of beat strength (strong-beat vs. weak-beat vs. non-beat) and stimulation type (sham vs. anodal vs. cathodal). Brain area (SMA, right cerebellum, right PMC or left PMC) and musical experience (high vs. low) were included as the between-subject factors, in order for Bayesian statistics to consider all combinations and interactions of factors to try to explain the data behaviour and the best model to fit the data. The best model represents a higher BF_M,_ or the change in likelihood of selecting a model prior to posterior data inclusion. The larger the BF, the more support for the model fitting the data. It is necessary to include the likelihood of each effect in the best model. From prior and posterior probabilities of including an effect across all models, BF_incl_ shows which factors are more likely to explain the data, with higher BF_incl_ showing stronger evidence for the hypothesis being tested. Analyses estimated the evidence for including each effect across matched models via estimating inclusion Bayes factor (BF_incl_) for each effect. Analyses also estimated the evidence for excluding each effect by estimating an exclusion Bayes factor (BF_excl_) for each effect. Where applicable, simple effects analyses were used to follow up interactions. Modified Jeffreys’s evidence categories for interpretation were taken as the standard for evaluation of the reported Bayes factors, in which the size of the Bayes factors are estimated as inconclusive (1–3), moderate (3–10), or strong (> 10) evidence for the hypotheses tested^45^.

##### Self-paced tapping task

For the self-paced tapping task, the intertap times were analyzed. The standard deviation of the intervals in each sequence was taken, and intervals that fell outside two standard deviations of the mean were removed from the analysis. Then, the coefficient of variation was calculated to measure the timing variability across each tap interval. A mixed-measures ANOVA was conducted on the coefficient of variation comparing the three stimulation sessions: sham, anodal and cathodal, with the brain area stimulated (SMA, right cerebellum, right PMC or left PMC) included as the between-subject factor.

## Results

### Musical training categorization

Participants were categorized into groups of high and low musical experience based on values of the Goldsmith MSI musical training subscale^35^. Those with a median score of 21 or more were assigned to the high musical experience group and lower than 21 to the low musical experience group, yielding 42 participants with high musical experience (range 21–44) and 39 with low musical experience (range 7–20). In the SMA group, the high/low split was 14/8; in the cerebellum group, 10/9; in the left PMC group, 8/11; and in the right PMC group, 10/11. Among participants with high musical experience, the average duration of music practice was 8 years. Ten participants indicated that they were actively practicing music, with an average of 4 h per week. Their musical backgrounds varied, including voice and different instruments such as drums, trumpet, piano, and guitar.

### Rhythm reproduction task

#### Proportion of correct trials

**Fig. 3:**
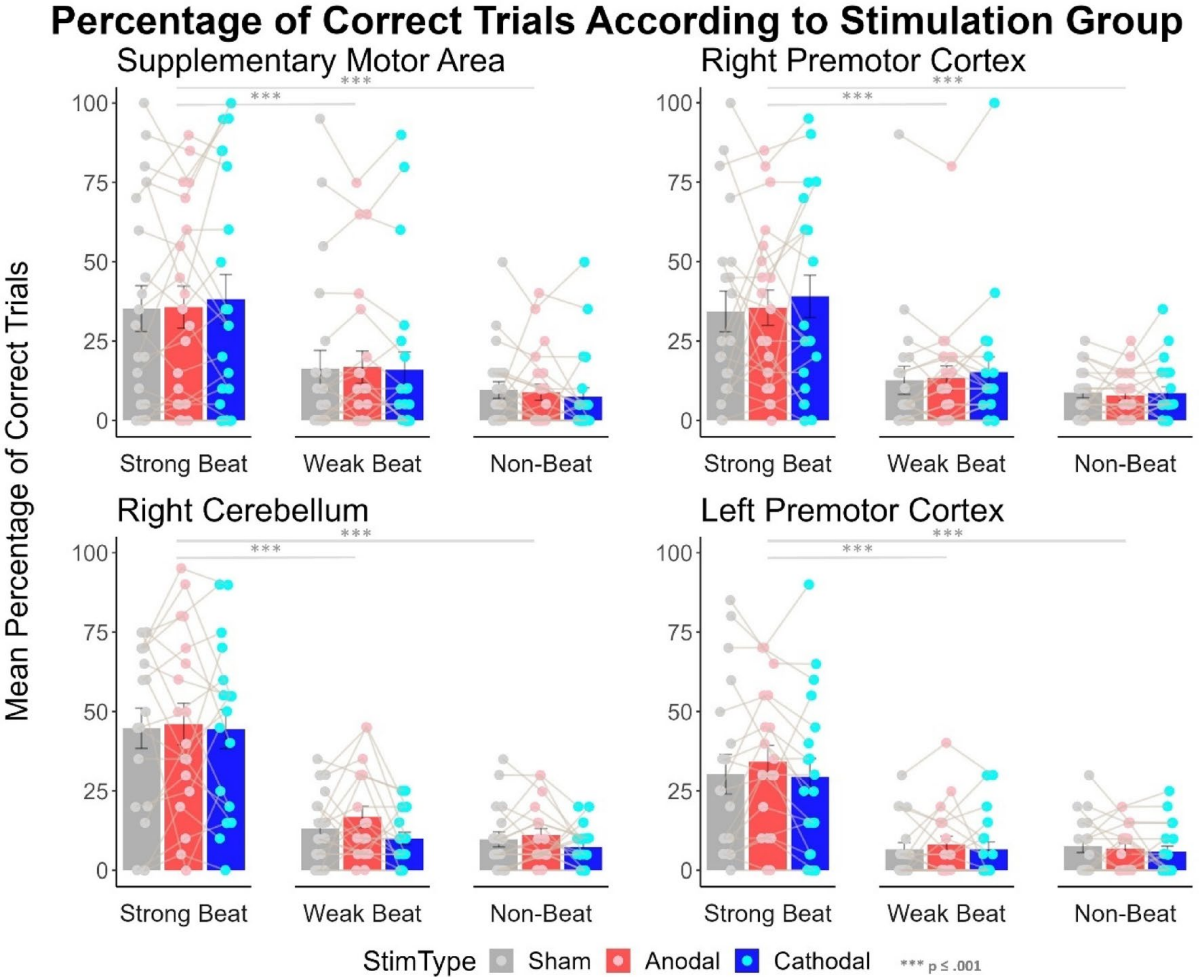
Response accuracy is determined according to the percentage of correct trials for the four groups: SMA, right PMC, right cerebellum, and left PMC. In each graph, bars are separated according to beat strength. Stimulation type is differentiated through the different colours; sham is represented in grey, anodal in red and cathodal in blue. Error bars represent the standard error of the mean. Individual data points are presented and connected through the grey line; values are jittered because of points that overlap. There are significant differences in accuracy when comparing beat strength but not when comparing different types of stimulation.

The proportion of correct trials is depicted in Fig. 3, with graphs separated according to the stimulation site. Mauchly’s test of sphericity indicated that the assumption of sphericity was violated for the following factors: beat strength and for the interaction between stimulation and beat strength (*p* < .05). We therefore used Greenhouse-Geisser sphericity correction for those factors only.

In the frequentist 4 × 2 × 3 × 3 mixed ANOVA, the four brain area groups did not differ in performance (*F*(3,73) = 0.76, *p* = .52, *ηp2* = 0.03). A main effect of music experience was observed (*F*(1,73) = 10.22, *p* < .01, *ηp2* = 0.12), as high musical experience participants (*M* = 24.76, *SD* = 25.99) performed better than low musical experience participants (*M* = 14.24, *SD* = 19.37).

**Fig. 4:**
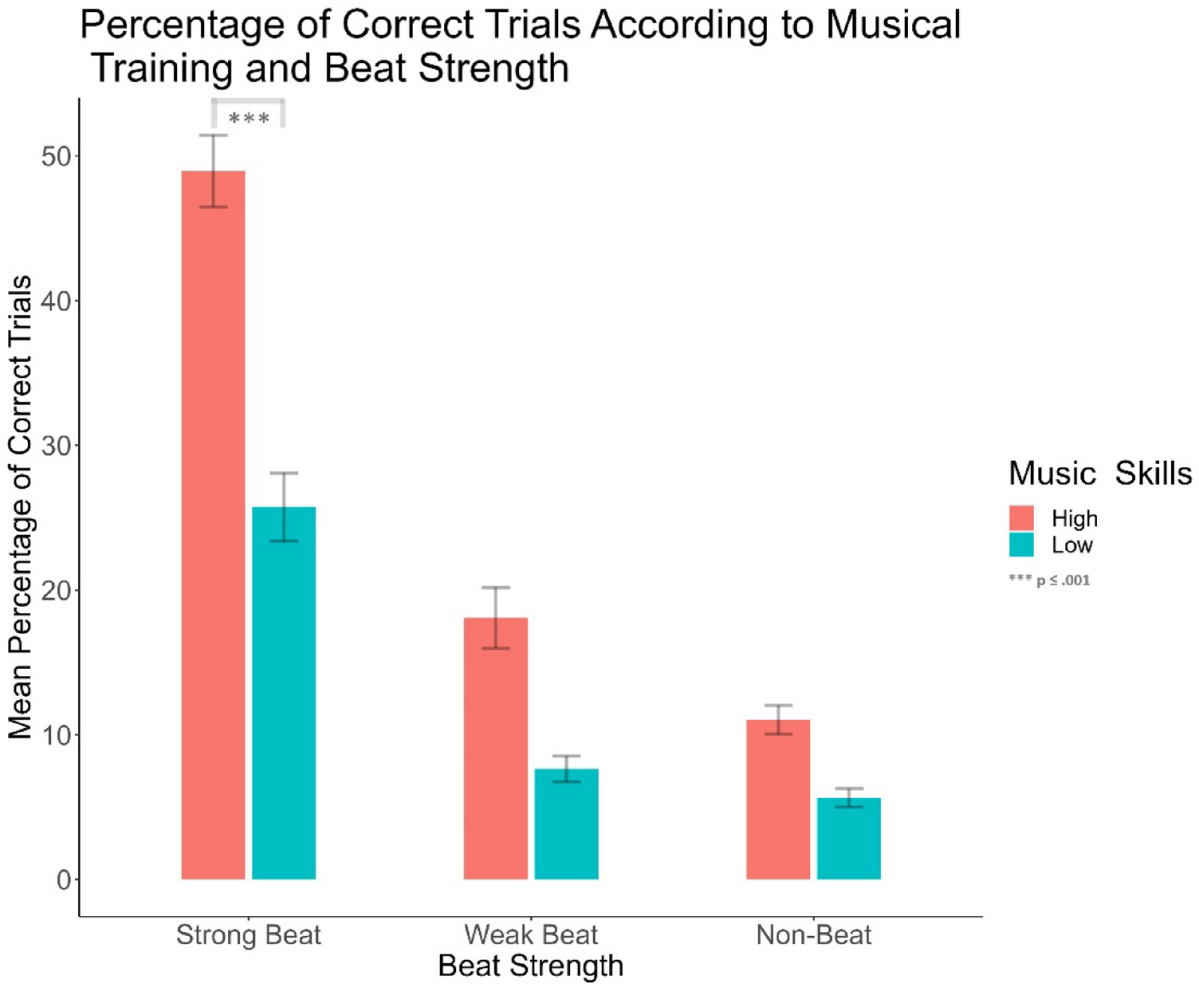
Response accuracy according to the percentage of correct trials comparing the interaction between music skills and beat strength. Bars are averaged over brain region group and stimulation type. Music skills are differentiated through the different colours; participants with high music skills are represented in coral colour, while participants with low music skills are represented by the cyan colour. Error bars represent the standard error of the mean. There are significant differences in accuracy when comparing participants with high and low music skills only for strong beat rhythms.

A significant main effect of beat strength was found (*F*(1.60, 117.12) = 118.54, *p* < .001, *ηp2* = 0.62). Post-hoc comparisons showed that strong-beat rhythms (*M* = 36.46, *SD* = 27.73) had a higher percentage of correct trials than weak- (*M* = 12.57, *SD* = 17.79, *t* = 12.19, *p* < .001) and non-beat rhythms (*M* = 8.12, *SD* = 8.36, *t* = 14.24, *p* < .001), and weak-beat rhythms had a higher percentage of correct trials than non-beat rhythms (*t* = 2.04, *p* = .04). Additionally, an interaction between beat strength and music experience was observed (*F*(1.60, 117.12) = 9.41, *p* < .001, *ηp2* = 0.11). Participants with high musical experience had significantly better reproduction performance for strong-beat rhythms (*M* = 47.12, *SD* = 26.62) than participants with low musical experience (*M* = 27.5, *SD* = 25.64, *t* = 4.94, *p* < .001), but not weak-beat rhythms (*M*_*high musical experience*_ = 16.67, *SD*_*high musical experience*_ = 21.71, *M*_*low musical experience*_ = 9.13, *SD*_*low musical experience*_ = 12.95, *t* = 2.11, *p* = .17) or non-beat rhythms (*M*_*high musical experience*_ = 10.50, *SD*_*high musical experience*_ = 9.65, *M*_*low musical experience*_ = 6.10, *SD*_*low musical experience*_ = 6.57, *t* = 1.04, *p* = .90). See Fig. 4.

There was no main effect of stimulation type (*F*(2, 146) = 0.77, *p* = .46, *ηp2* = 0.01). No interaction between stimulation type and beat strength was observed (*F*(2.97, 217.14) = 0.82, *p* = .48, *ηp2* = 0.01), nor between stimulation type and brain area (*F*(6, 146) = 1.29, *p* = .26, *ηp2* = 0.05) nor between the stimulation type and music experience (*F*(2, 146) = 0.14, *p* = .87, *ηp2* = 0.002).

#### Bayesian statistics

The best-fitting model for our data included the combination of beat strength (Beat) + brain area (Brain) + musical experience (Music) + interaction of beat strength (Beat) with brain area (Brain) + interaction of beat strength (Beat) with musical experience (Music), with *BF*_*M*_ = 190.56.

When analyzing the effects and including all the factors (Table 2), beat strength is very likely to explain our data (*BF*_*incl*_ = 2.67e^92^), followed by the interaction between beat strength and musical experience (*BF*_*incl*_ = 1.14e^9^), and by the interaction between beat strength and the brain region group (*BF*_*incl*_ = 58.07). Musical experience is also likely to explain the data (*BF*_*incl*_ = 20.54).

**Table 2:** Bayesian analysis of the percentage of correct trials effects.

Effects	*P*(incl)	*P*(incl|data)	*P*(excl|data)	BF _incl_
Stim	0.11	0.022	0.99	0.023
**Beat**	0.11	2.54e^−10^	9.49e^−103^	2.67e^+92^
Brain	0.11	0.009	0.058	0.15
**Music**	0.11	5.64e^−10^	2.75e^−11^	20.54
Stim ✻ Beat	0.30	1.81e^−4^	0.023	0.008
Stim ✻ Brain	0.30	2.25e^−4^	0.020	0.011
Stim ✻ Music	0.30	7.77e^−4^	0.023	0.034
**Beat** ✻ **Brain**	0.30	0.74	0.013	58.07
**Beat** ✻ **Music**	0.30	0.81	7.09e^−10^	1.14e^+9^
Group ✻ Music	0.30	0.20	0.55	0.36
Stim ✻ Beat ✻ Brain	0.11	2.44e^−9^	2.96e^−6^	8.24e^−4^
Stim ✻ Beat ✻ Music	0.11	1.23e^−7^	7.75e^−6^	0.016
Stim ✻ Brain ✻ Music	0.11	4.17e^−8^	3.42e^−6^	0.012
Beat ✻ Brain ✻ Music	0.11	0.19	0.19	1.01
Stim ✻ Beat ✻ Brain ✻ Music	0.006	4.24e^−19^	4.95e^−17^	0.009

Bayesian Analysis of the Percentage of Correct Trials Effects. Stim = stimulation type (sham, anodal, cathodal); Beat = beat strength (strong, weak, non-beat); Brain = brain area stimulated (SMA, right cerebellum, left PMC or right PMC); Music = musical experience (high or low); * = interactions. Factors with strong evidence (BF > 10) are in bold.

Table 2. Bayesian Analysis of the Percentage of Correct Trials Effects. Stim = stimulation type (sham, anodal, cathodal); Beat = beat strength (strong, weak, non-beat); Brain = brain area stimulated (SMA, right cerebellum, left PMC or right PMC); Music = musical experience (high or low); * = interactions. Factors with strong evidence (BF > 10) are in bold.

#### Proportional average error

**Fig. 5:**
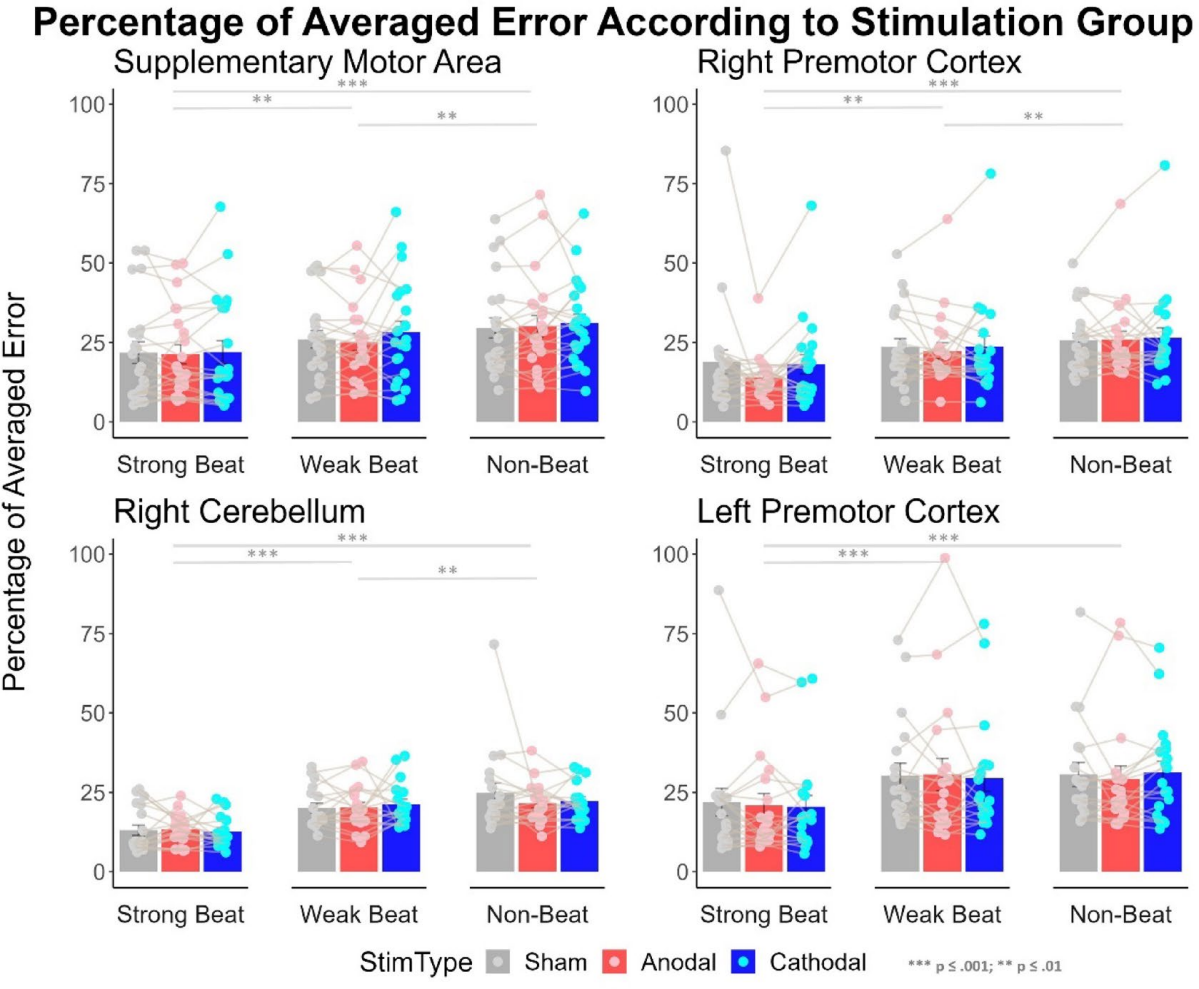
Proportion for the averaged error for the four groups (SMA, right PMC, right cerebellum, and left PMC). Perfect performance would result in 0% of averaged error; the bigger the values, the more averaged error is shown. The average error is separated according to beat strength. Stimulation type is differentiated through the different colours; sham is represented in grey, anodal in red and cathodal in blue. Error bars represent the standard error of the mean. Individual data points are presented and connected through the grey line; values are jittered because of points that overlap. There are significant differences in the averaged error when comparing beat strength but not when comparing different types of stimulation.

The proportion of average error is depicted in Fig. 5, with graphs separated according to the stimulation site. Mauchly’s test of sphericity indicated that the assumption of sphericity was violated for beat strength and for the interaction between stimulation and beat strength (*p* < .05). We therefore used Greenhouse-Geisser sphericity correction for those factors only.

In the frequentist 4 × 2 × 3 × 3 mixed ANOVA, the four brain area groups did not differ in performance (*F*(3,73) = 2.47, *p* = .07, *ηp2* = 0.09). However, a main effect of music experience was observed (*F*(1,73) = 4.38, *p* = .04, *ηp2* = 0.06), as high musical experience participants (*M* = 20.40, *SD* = 12.29) had less average reproduction error than low musical experience participants (*M* = 26.13, *SD* = 13.96).

A significant main effect of beat strength was found (*F*(1.76, 128.48) = 137.60, *p* < .001, *ηp2* = 0.65). Post-hoc comparisons showed that strong-beat rhythms (*M* = 18.25, *SD* = 13.11) had lower average error than weak- (*M* = 24.91, *SD* = 13.23, *t* = 11.67, *p* < .001) and non-beat rhythms (*M* = 27.37, *SD* = 12.64, *t* = 16.05, *p* < .001), and weak-beat rhythms had a significantly lower average error than non-beat rhythms (*t* = 4.38, *p* < .001).

There was no main effect of stimulation type (*F*(2, 146) = 0.75, *p* = .48, *ηp2* = 0.01). No interaction between the stimulation type and beat strength was observed (*F*(2.96, 216.01) = 0.31, *p* = .81, *ηp2* = 0.004), nor between the stimulation type and brain area being stimulated (*F*(6, 146) = 0.45, *p* = .84, *ηp2* = 0.02) nor between the stimulation type and music experience (*F*(2,146) = 1.36, *p* = .26, *ηp2* = 0.02).

#### Self-Paced tapping task

**Fig. 6:**
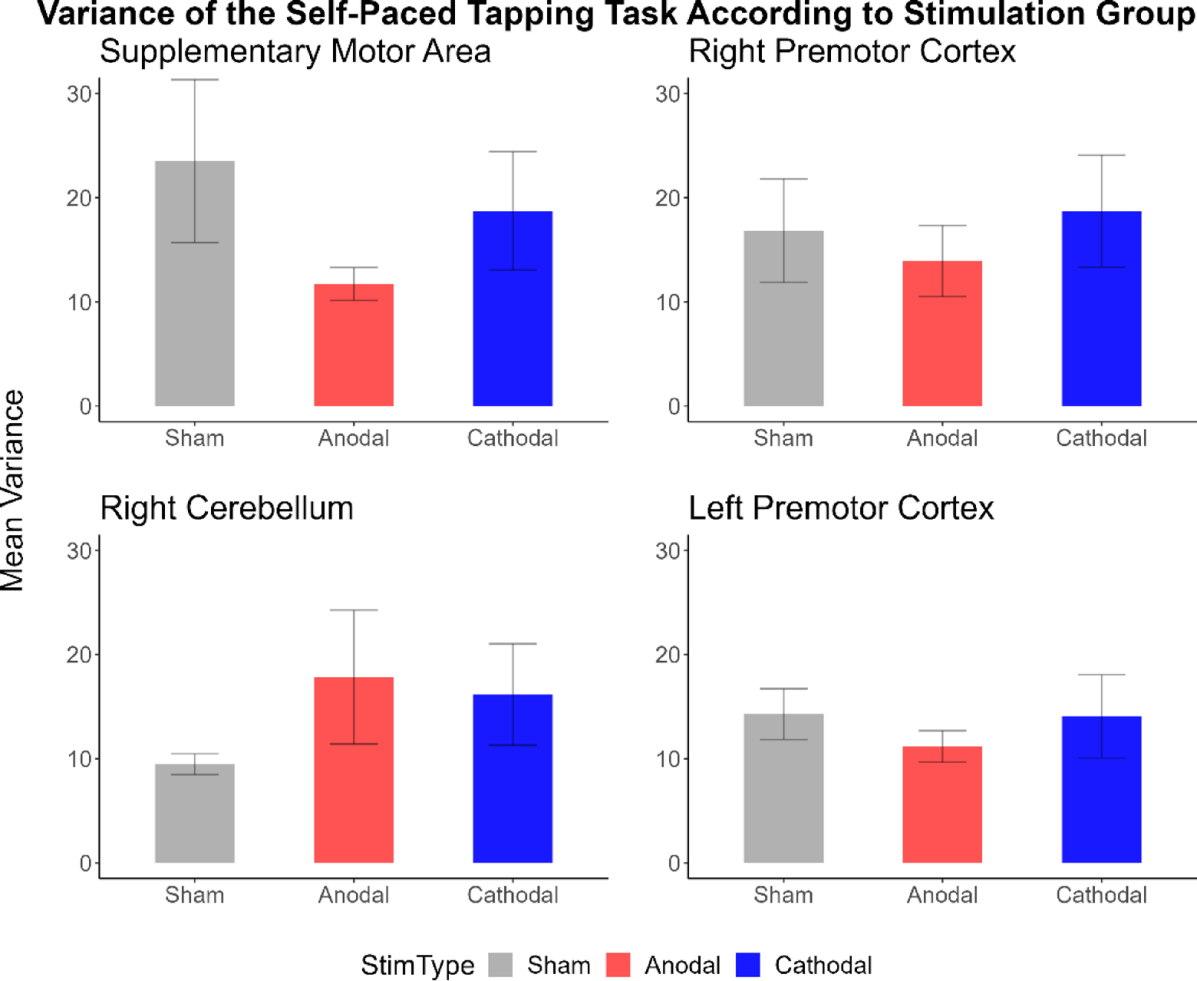
Self-paced tapping task. Mean coefficient of variance for the four groups (SMA, right PMC, right cerebellum, and left PMC). Sham is in grey, anodal in red and cathodal in blue. No significant difference was seen between stimulation types and groups.

The four brain stimulation groups did not differ in the variability of self-paced tapping (*F*(3,73) = 0.73, *p* = .53, *ηp2* = 0.03). There was no main effect of stimulation when comparing sham vs. anodal vs. cathodal sessions (*F*(2,146) = 0.53, *p* = .56, *ηp2* = 0.01). No interaction between stimulation type and musical experience was seen (*F*(2,146) = 0.01, *p* = .99, *ηp2* = 1.78e^−4^), or between stimulation type and brain area being stimulated (*F*(6,146) = 0.90, *p* = .50, *ηp2* = 0.04), nor between stimulation type, brain area being stimulated and music skills (*F*(6,146) = 1.38, *p* = .22, *ηp2* = 0.05). See Fig. 6.

#### Blindness towards stimulation

Each participant completed three sessions: two active (anodal and cathodal) and one sham. After completing all sessions, participants guessed whether each session was active or sham. Of the 81 participants, 31 (38%) correctly identified all three sessions, 34 (42%) correctly identified two sessions, 13 (16%) correctly identified one session, and 3 (4%) did not correctly identify any sessions. Participants also rated their confidence in their guesses on a scale from 1 (completely unsure) to 10 (completely sure), with an overall average confidence score of 2.98 across the three sessions.

## Discussion

In this study, we investigated the causal role of four brain areas: SMA, right cerebellum, left PMC, and right PMC, in rhythm reproduction. We hypothesized that modulating excitability in the SMA would influence the ability to reproduce strong-beat rhythms, while altering the excitability of the cerebellum and PMC would influence the ability to reproduce all types of rhythms, or weak and non-beat rhythms, when compared to the SMA stimulation. Contrary to our main hypotheses, modulation of SMA, PMC, and cerebellum had no effect on reproduction accuracy for any of the rhythm types nor on self-paced tapping variability. As predicted, participants, especially those with musical training, performed strong-beat rhythms better than weak- and non-beat rhythms, replicating previous findings^5,46,47^. Bayesian analysis confirmed that there was strong evidence for beat strength and musical experience affecting rhythm reproduction performance, and that there was little evidence for the tDCS effects on the reproduction of rhythms. The Bayesian analysis did show evidence of an interaction between beat strength and the brain area stimulated (irrespective of polarity), but, as the brain area that was stimulated was manipulated between groups, this may be due to the sample variability.

### Motor brain regions and their roles in beat and time perception

Rhythm processing and beat perception rely on a distributed and interactive network of motor and auditory regions, rather than isolated brain areas. Two primary timing mechanisms have been proposed: beat-based timing, where intervals are encoded relative to a periodic beat^18,21,48^, and absolute timing, where each interval is processed independently, like a stopwatch^31^. While beat-based timing is commonly linked to the SMA and basal ganglia^5,8,13,18,49^, and absolute timing to the cerebellum^20,31^, growing evidence suggests that these systems overlap and interact dynamically. The PMC, for example, has been implicated in both timing mechanisms, depending on task demands^7,8,50^. Similarly, the cerebellum has also been implicated in beat perception when rhythms are less regular^7,23^, showing its flexible role in rhythm processing.

This complexity may explain why tDCS over SMA, PMC, and cerebellum did not produce the predicted effects on rhythm reproduction. While previous research demonstrated that SMA stimulation affected rhythm discrimination^23^, the absence of effects on rhythm reproduction suggests that the role of the stimulated regions may be more pronounced in perceptual timing tasks. Given that rhythm reproduction involves both perceptual and motor processes, it is possible that additional motor regions contributed to performance, making it less sensitive to focal tDCS modulation. Alternatively, the task demands may have engaged compensatory mechanisms across the broader timing network, reducing the likelihood of detecting localized effects.

### Supplementary motor area

The main objective of our study was to extend the findings of a previous tDCS study on rhythm discrimination^23^ that showed SMA stimulation affected rhythm discrimination, with anodal stimulation improving performance and cathodal stimulation worsening it. Interestingly, strong and weak beat rhythms were similarly affected by stimulation, suggesting the task-relevant role of the SMA did not depend on beat strength. In that study, the weak-beat rhythms may not have been irregular enough for participants to rely on the non-beat-based timing system – weak-beat rhythms can still be encoded relative to an underlying beat, even if it is not felt as strongly as in strong-beat rhythms. Therefore, in the current study we included non-beat rhythms, in which no beat can be perceived. However, we observed no effects of SMA stimulation on rhythm reproduction for any rhythm type. Future research could test the discrimination of non-beat rhythms and examine whether SMA stimulation affects beat-based timing for strong- and weak-beat rhythms but not non-beat rhythms.

The absence of a causal effect within the SMA was not predicted, particularly in light of the substantial body of research that has consistently highlighted the SMA’s involvement in rhythm and beat perception^5,16,23^. The lack of effect during rhythm reproduction may indicate that the SMA’s role in timing is more evident in perceptual tasks. As tapping along to a rhythm improves timing perception^51^, it’s possible that the movement involved in reproducing the rhythm overrides any effect of stimulation. The need to produce accurately timed movement sequences may recruit additional motor brain areas, masking potentially subtle effects on timing produced by SMA stimulation. While the SMA is implicated in timing, the simultaneous involvement of other brain regions in motor control makes it challenging to pinpoint the exact impact of SMA stimulation on timing in the context of movement.

Striato-thalamo-cortical loops, of which the SMA are a part, are essential for temporal predictions and the production and discrimination of time intervals^52,53^. The SMA is thought to anticipate the next beat in a sequence and send direct signals to the dorsal striatum, which then creates representations of beat cycle intervals, and activates different or inactive SMA neural subpopulations that would be activated in response to the current beat cycle via the thalamus^15^. The SMA primarily supports beat-based timing sequences by planning when the next beat should occur within the sequence.

Furthermore, the SMA is involved in the internal generation of timing during synchronization-continuation tasks^15,54–56^. This aligns with the idea that the SMA is involved in generating the next beat in a sequence^15^. The SMA activates more during continuation than synchronization^54,55^, and patients with SMA lesions are impaired at continuation but not synchronization^54,56^. This suggests that the SMA is particularly crucial for tasks where timing is internally generated^57^. Nonetheless, we did not find an effect of stimulation here.

### Cerebellum

We did not find an effect of tDCS applied to the right cerebellum on rhythm reproduction. Previous studies have demonstrated cerebellum tDCS effects on various cognitive and motor tasks, including reaction time, working memory, motor learning, and motor memory^58–60^. However, null effects of cerebellar tDCS are common in tasks involving associative learning, working memory, implicit learning, motor adaptation, and cognitive function^61–64^. These conflicting results^65,66^ may be due to anatomical differences between the cerebellum and the cerebral cortex, as it is organized differently from the cortex^67^. Consequently, polarity differences (anodal versus cathodal) in tDCS effects are less predictable, making it challenging to anticipate the direction of behavioural changes^39,65^. In our study, however, we did not observe any effect of stimulation, anodal or cathodal.

Although our study found a null result for cerebellar stimulation, it does not diminish the importance of the cerebellum in motor control, movement precision^68,69^, and absolute timing^18,20,29,32^. It is possible we did not effectively target the appropriate region of the cerebellum with our tDCS, and future work could use neuroimaging to enhance the targeting of the stimulation. However, this same montage did produce effects on rhythm discrimination in previous work^23^, albeit in the strong-beat condition only, which was also contrary to evidence of selective cerebellar involvement in absolute timing.

### Premotor cortex

For PMC, we expected to either see tDCS effects across all rhythms, or more specifically, for weak- and non-beat rhythms, consistent with a role in absolute timing^8^. No effect of anodal or cathodal stimulation was seen for either left or right premotor cortices.

There is evidence linking the PMC to both beat-based and non-beat based, or absolute, timing, particularly in studies of rhythm synchronization^6,50,70,71^. Different subregions of the PMC have been found to serve distinct roles in rhythm synchronization. For example, the ventral PMC is activated when participants listen to rhythms before synchronizing to them, while the dorsal PMC is responsive during synchronization, especially with more complex rhythms. The mid-PMC, along with the SMA and cerebellum, is activated when participants listen to rhythms without the intention of performing motor actions^70^. Additionally, the dorsal PMC has been shown to exhibit increased activation when the beat of a rhythm is made more salient^50^. These findings highlight the involvement of the PMC in motor-auditory interactions during movement sequencing.

The null effects observed in our study are not entirely surprising considering the PMC’s general role in motor-time synchronization, and its activation in studies where no motor output, such as rhythm reproduction, is required. It is possible that our task failed to incorporate a crucial aspect of the PMC’s function, namely motor synchronization to an auditory stimulus. Participants did not have the opportunity to synchronize their movements to the rhythmic sequences but rather had to reproduce each rhythm from memory after hearing it multiple times. Furthermore, PMC stimulation did not have a reliable effect on rhythm discrimination in previous work^23^,suggesting that the PMC may not play a significant or direct role in rhythm discrimination or reproduction itself. A rhythm synchronization paradigm may show the effects of PMC stimulation on timing performance.

## Limitations

One limitation of our study was the absence of a control task that has previously been shown to yield consistent results for SMA stimulation. A control task that is consistently and predictably affected by stimulation has the benefit of adding a ground truth to stimulation studies – they tell us whether (and in which subjects) tDCS successfully modulated brain responses. Here, we tested whether self-paced tapping could be used as a control task but failed to demonstrate any effects of stimulation. While this could suggest that tDCS over the four brain areas studied here does not affect self-paced rhythm or motor output, it also means that we cannot confirm our predictions or validate the efficacy of stimulation in the sample. To address this, future research could explore different control tasks tailored to each specific brain area. If stimulation produces consistent effects in these control tasks but not in a rhythm reproduction task, it would support the conclusion that these brain areas do not have a causal role in beat perception during rhythm reproduction.

Another limitation of our study is the difficulty in modulating focal brain activity using tDCS. Unlike TMS, which allows for more focal modulation of cortical areas^72^, tDCS influences brain function in a more diffuse and polarity-dependent manner, and relies on applying tDCS during task performance to selectively modulate relevant circuits^73^. While the stimulation itself is relatively non-specific, its interaction with the task-induced neural activity results in a selective effect on the neurons that are actively engaged in the task. Whilst prior TMS research using continuous theta burst stimulation (inhibitory stimulation) to the left SMA and left posterior parietal cortex has provided evidence for causal contributions of the dorsal auditory stream to phase perception in beat-based timing—the ability to detect when a beat occurs earlier or later than expected in the context of music—the same study found no effect of SMA downregulation on beat-based timing^74^. This aligns with our findings, suggesting that the SMA may play a context-dependent role in rhythm perception, and also that stimulus differences (music versus rhythms) may matter. Future studies could consider complementary approaches, such as TMS or neuroimaging-guided tDCS, or comparing different types of beat-based stimuli in the same study.

Finally, another important limitation is the use of a single-blind design where only participants were unaware of the type of stimulation received. Blinding tDCS is challenging due to the tingling and itching sensations often experienced by participants^75,76^. Despite this limitation, our study was good in maintaining participants’ blindness towards the type of stimulation given the lower confidence ratings of the type of stimulation received.

## General conclusions

Overall, in contrast to findings of tDCS stimulation of SMA influencing rhythm discrimination^23^ neither anodal nor cathodal tDCS stimulation affected rhythm reproduction accuracy or variability of self-paced tapping. Consistent with prior work, there were effects of beat strength, with strong-beat rhythms reproduced more accurately than weak- and non-beat rhythms. It is possible that the requirement for overt movement mitigated more subtle effects of stimulation on rhythm perception. Similarly, effects of stimulation on rhythm discrimination were seen for the cerebellum, but not during reproduction here. The roles of motor brain regions in rhythm perception and production, therefore, may be easier to observe with tDCS when more perceptual, rather than motor, tasks are used. Importantly, our study employed identical stimulation montages, stimuli, and equipment as a prior rhythm discrimination study that did find polarity-dependent effects of SMA stimulation on rhythm processing^23^. Given methodological consistency, our null results must be interpreted in the context of these prior findings. The findings suggest a distinction between the effects of SMA excitability on rhythm discrimination versus reproduction. Our results highlight task-specific neural processing differences, suggesting that motor execution requirements may swamp effects of motor area stimulation that are otherwise observed in perceptual tasks.

## Data Availability

The data supporting the findings of this study are available in OSF at osf.io/3wvq9, under the DOI 10.17605/OSF.IO/3WVQ9. The dataset includes anonymized information to ensure participant privacy and compliance with ethical guidelines. For additional information or specific data requests, please contact the corresponding author.
